# Maintaining Essential Nutrition Services to Underfive Children in Yemen: A Programmatic Adaptation Amidst the COVID-19 Pandemic

**DOI:** 10.3390/children8050350

**Published:** 2021-04-28

**Authors:** Ferima Coulibaly-Zerbo, Ayoub Al-Jawaldeh, Zita C. Weise Prinzo, Marina Adrianopoli, Eshrak Naji Mohammed Al-Falahi, Shafekah Alahnoumy, Nosheen Mohsan Usman, Fanda Ahmed Moqbel, Latifah Abbas Ali, Mohammed Shroh, Ensegam Mohammed Al-Sakkaf, Abdulrazzaq Musaed, Maison Al-Sakkaf, Mohammed Dahnan, Fahim Al-Hakimi, Doa Kutbi Omer, Moatsim Hael, Lara Nasreddine

**Affiliations:** 1 World Health Organization (WHO), Sana’a 543, Yemen; zerbof@who.int (F.C.-Z.); madrianopoli@who.int (M.A.); alfalahie@who.int (E.N.M.A.-F.); alahnoumys@who.int (S.A.); moqbelf@who.int (F.A.M.); alilat@who.int (L.A.A.); shrohm@who.int (M.S.); alsakkafe@who.int (E.M.A.-S.); musaeda@who.int (A.M.); alsakkafm@who.int (M.A.-S.); dahnanm@who.int (M.D.); alhakimif@who.int (F.A.-H.); kutbid@who.int (D.K.O.); haelm@who.int (M.H.); 2 Regional Office for the Eastern Mediterranean (EMRO), World Health Organization (WHO), Cairo 7608, Egypt; aljawaldeha@who.int; 3 Department of Nutrition and Food Safety, World Health Organization (WHO), Avenue Appia 20, 1211 Genève 27, Switzerland; weiseprinzoz@who.int; 4 Water, Sanitation and Environmental Health Department, World Health Organization (WHO), Sana’a 543, Yemen; usmann@who.int; 5 Nutrition and Food Sciences Department, Faculty of Agriculture and Food Sciences, American University of Beirut, Beirut 11-0236, Lebanon

**Keywords:** COVID-19, nutrition, adaptation, under-five children, Yemen

## Abstract

The World Health Organization (WHO) acknowledged that, as health services divert their attention to the COVID-19 pandemic, the delivery of essential nutrition services may be compromised. This impact may be more pronounced in the context of humanitarian crises, such as the one currently unfolding in Yemen. In line with Pillar 9 of the WHO’s COVID-19 Strategic Preparedness and Response Plan, this paper reports on the nutrition program adaptations in Yemen to maintain the delivery of essential nutrition services to under-five children. The process of adaptation focused on the services provided within the nutrition surveillance system (NSS), therapeutic feeding centers (TFC), and isolation units (IU). It was conducted in five steps: (1) situation analysis; (2) development of guidance documents; (3) consultation process; (4) capacity-building programs; and (5) incorporation of programmatic adaptation within nutrition services. As of September 2020, NSS, TFC, and IUs services have shifted their standard operating procedures in line with the context-specific adaptations. The process described in this paper may serve as a case-study for other countries that intend to undertake similar adaptations in their nutrition program to contribute to the implementation of the WHO response plan and maintain the delivery of essential nutrition services to children.

## 1. Introduction

The year 2020 has witnessed the spread of the Coronavirus disease 2019 (COVID-19) leading to a profound universal crisis, with unprecedented reach and proportions. On 30 January 2020, the World Health Organization (WHO) described the COVID-19 outbreak “as a public health emergency of international concern under the International Health Regulations” [1], and on 3 March 2020, the WHO announced COVID-19 outbreak as a pandemic [2]. The WHO Director-General called for the activation of the United Nations (UN) crisis management policy and the establishment of a crisis management team to coordinate the UN’s system-wide scale up and assist countries in their preparedness and response plan [1]. It was recommended that national authorities develop and update their COVID-19 country-specific plans across the major pillars of COVID-19 preparedness and response, where Pillar 9 targeted the maintenance of essential health services and systems amidst the outbreak [1].

At the global level, health systems have been challenged by the escalating demand for care of COVID-19 patients, compounded by fear, misinformation, and restrictions on movement that disturb or interrupt health care delivery, for all conditions [3]. Whenever health systems are overwhelmed and people’s access to needed care is compromised, both direct mortality (from the outbreak) and indirect mortality (from preventable and treatable conditions and diseases) would increase considerably [4,5,6]. These impacts may be more pronounced in low-capacity and humanitarian settings, due to their already fragile health systems [1]. In this context, the humanitarian crisis in Yemen remains the worst in the world, driven by conflict, diseases, economic collapse and the breakdown of public institutions and services [7]. A staggering 80% of the population requires humanitarian assistance. Over 17.9 million people in Yemen are in dire need of health assistance, of which 14.3 million are in acute need [7]. Only 51% of health facilities are fully functional [7]. Poor vaccination coverage, critical water shortages and related poor hygiene, a collapse of sanitation systems, and massive population displacement have given way to a surge in the spread of diseases [8]. The recent cholera and diphtheria outbreaks particularly highlight the detrimental impact of a failing health system.

Since the escalation of the conflict in 2015 in Yemen, rates of malnutrition have grown alarmingly, with famine remaining a persistent menace in the country. In fact, and as per the 2020 Nobel Peace prize announcement, “the link between hunger and armed conflict is a vicious circle: war and conflict can cause food insecurity and hunger, just as hunger and food insecurity can cause latent conflicts to flare up and trigger the use of violence” [9]. The combination of violent conflict and the COVID-19 pandemic “has led to further aggravation and dramatic increases in “the number of people living on the brink of starvation” [9]. Two-thirds of all Yemenis are currently hungry [10], and in 2021, 2,254,663 Yemeni children are estimated to suffer from acute malnutrition, with nearly 400,000 suffering from severe acute malnutrition (SAM). Of these, up to 10% may end up with severe medical complications [11]. These children need immediate access to nutrition and health services to survive. A more recent analysis showed that 45% (13.5 million) of the population in Yemen is facing high acute food insecurity [12].

In this emergency context, and in line with the humanitarian reform agenda, the coordination of the response in Yemen adopted the cluster approach as one of the elements to enhance predictability, accountability, and partnership [13]. This approach revolves around nine clusters, where each cluster consists of groups of humanitarian organizations, both United-nations (UN) and non-UN, in each of the main sectors of humanitarian action, including the nutrition cluster and the health cluster [13]. As such, and in response to the alarming nutritional crisis that was unfolding in the country, WHO Yemen has launched in 2016, in partnership with the nutrition cluster and the Ministry of Public Health and Population (MOPHP) the Nutrition Program under the WHO’s Health Emergency Program (WHE) [14,15,16]. The overall goal of this program is to improve child nutritional status through identification, referral, and treatment of children with wasting, to reduce morbidity and mortality associated with SAM, while monitoring the nutritional situation for timely and appropriate response to all forms of undernutrition, with special focus on wasting. The WHE Nutrition Program therefore developed around two key pillars: (a) inpatient management of children suffering from SAM and related medical complications and (b) nutrition surveillance system (NSS). At the onset of the COVID-19 outbreak in the country (10 April 2020), the nutrition cluster partners have reported a number of worrying effects on the program’s implementation, namely changes in the population’s healthcare seeking behaviors, due to the fear of infection, movement restrictions for mobile teams, increased supplies’ costs, and the repurposing of nutrition centers into COVID-19 designated facilities [17]. These factors have affected access to service amongst the most vulnerable population, in a country where people, communities, and systems are dramatically characterized by low resilience.

In its interim guidance document on the maintenance of essential health services in the COVID-19 context [3], the WHO acknowledged that, as health services divert their attention to the COVID-19 pandemic, the delivery of essential nutrition services are threatened, along with the surveillance of at-risk populations [3]. This highlights the need for adaptation at the level of nutrition services and programs, in order to prevent increases in malnutrition-related morbidity and mortality [3]. The adaptations that need to be made depend on the phase of the pandemic, the response, and the country context, including the food security situation and burden of malnutrition. In areas with a high burden of malnutrition, essential nutrition actions for vulnerable population groups, such as under-five children (U5C), should be maintained throughout all stages of the response. Nutrition services should therefore be adapted to ensure coordination of nutrition activities, enhanced service utilization, optimal work implementation, as well as its scaling up. In direct contribution to Pillar 9 within the COVID-19 strategic preparedness and response plan [1], the WHO Yemen office has undertaken strategic adaptations to ensure access to safe nutrition services during the COVID-19 pandemic and maintain essential nutrition services in the health sector. While drawing on the unique local context of Yemen, this paper aims to describe the process adopted for the nutrition programmatic adaptation, and delineate key considerations addressed within this adaptation as well as aspects related to its monitoring. This paper may serve as a case study for other countries that intend to undertake similar adaptations in their nutrition program to contribute to the implementation of the WHO response plan to the COVID-19 pandemic and maintain the delivery of essential nutrition services in the health sector.

## 2. Understanding the Yemeni Context

### 2.1. COVID-19 Situation in Yemen

The COVID-19 outbreak was declared in Yemen on 10 April 2020 [18]. The Yemen health cluster is actively responding to the COVID-19 pandemic in addition to the health consequences of the conflict and the severe economic crisis in the country [19]. Serving as the Health Cluster lead agency, the WHO is playing a vital role in ensuring cooperation and coordination between the Health Cluster partners on the various humanitarian health interventions [18].

As of 28 February 2021, the total number of COVID-19 cases that was reported by national authorities for 11 governorates of Yemen is of 2273, with 632 deaths [20]. The real number of cases is however suspected to be much higher than this, as no official line lists are received from Northern governorates and with the testing capacity across the country being still low: this suggests that community spread may be occurring silently, unmeasured, and uncontrolled despite efforts to support the preparedness and response and maintain high vigilance [19]. In addition, delays in seeking treatment because of stigma, difficulty in accessing treatment facilities and the perceived risk of seeking care are amongst the reasons behind the low number of the reported cases [19]. Health partners continue to work towards increasing COVID-19 surveillance, deploying dedicated staff, and enhancing intensive care unit (ICU) capacity, assessing the virus’s impact on routine priority health programs, and developing and improving community-based messaging to foster behavioral change [19], although to date, these efforts have had minimal or insufficient impact on the COVID-19 situation in the country.

### 2.2. Nutrition Situation of U5C in Yemen

Yemen’s civil war started in 2014, when Houthi insurgents took control of Yemen’s capital Sana’a, and made demands for a new government [21]. In 2016, the Houthis announced the formation of a “political council” that governs Sana’a, and much of northern Yemen. The intervention of regional powers in the conflict continues to draw the country into a broader divide. In the meantime, the ongoing conflict is taking a heavy toll on civilians’ lives, rendering Yemen the world’s worst humanitarian crisis [21].

It has been estimated that 20 million people are suffering from food insecurity and around a quarter of a million are afflicted with starvation [15] in Yemen. Amidst this humanitarian crisis, young children are described as the “sickest of the sick” [15]. In 2020, 2.1 million Yemeni children were reported to suffer from acute malnutrition, with nearly 320,000 suffering from SAM [22]. Almost a third of children aged 6–59 months are regularly found to be suffering from wasting, and around two-thirds show signs of stunting [16]. In December 2020, more than 41,000 children attended screening sessions in the nutrition surveillance sites, and 22% were found to be suffering from wasting and were referred to nutrition treatment programs [15]. In addition, half of all children screened in December 2020 were found to be underweight [23]. The projections for the year 2021 are that more than 2.25 million children aged 0 to 59 months will suffer from acute malnutrition in the course of the year in Yemen [17].

Factors contributing to the high burden of child malnutrition include the high prevalence of communicable diseases including diarrheal diseases and malaria or fever, high levels of acute food insecurity, poor infant and young child feeding practices, as well as poor water, sanitation, and hygiene [17]. In addition, access to nutrition and health services may have been compromised by the ongoing conflict in several zones, as well as the decline in access and utilization of health and nutrition services as a result of the COVID-19 pandemic [17,24]. The direct and indirect effect of COVID-19, such as the reduction in remittances, decreased access to markets, challenges in maintaining employment, compounded by economic shocks, such as delayed salary payments and conflict, have contributed to a deterioration of child acute malnutrition [17,24].

### 2.3. WHE Nutrition Program in Yemen

To detect early cases of malnutrition in U5C, the WHO has set up, in 2018, a nationwide nutrition surveillance system. Through a network of nutrition surveillance sites located in district hospitals across the country, health workers (HW) are able to find and refer children with wasting, as well as those suffering from stunting. In alignment with the Nutrition Cluster top priority for 2020 [25] to reduce acute malnutrition amongst highly vulnerable populations, and to significantly contribute to the Cluster’s first line response to identify and refer children with acute malnutrition to treatment programs, the WHO has established, between 2019 and 2020, over 140 nutrition surveillance sites across 21 governorates, with plans in place to scale up the system to all functioning hospitals. In 2020, approximately 307,000 U5C were screened for malnutrition across the nutrition surveillance sites established in Yemen [15].

To increase access to treatment for severely malnourished children with medical complications, the WHO supports 99 therapeutic feeding centers (TFC) across the country, with plans to scale up to 114 in 2021. Support comes in the form of equipment, furniture, and kits to treat severe acute malnutrition, in addition to providing staff training and incentives for HWs. In 2020, the WHO had supported TFCs in 222 of Yemen’s priority districts covering all the 22 governorates. These TFCs provide free-of-charge treatment, milk, and medicines to children affected with SAM with medical complication, as well as health education to families. Available figures indicate that the quality of service provided by these centers improved steadily in 2020, with 91% children being reported as cured of acute malnutrition with complications [16].

## 3. Adaptation of the Nutrition Program: The Process

**Figure 1:**
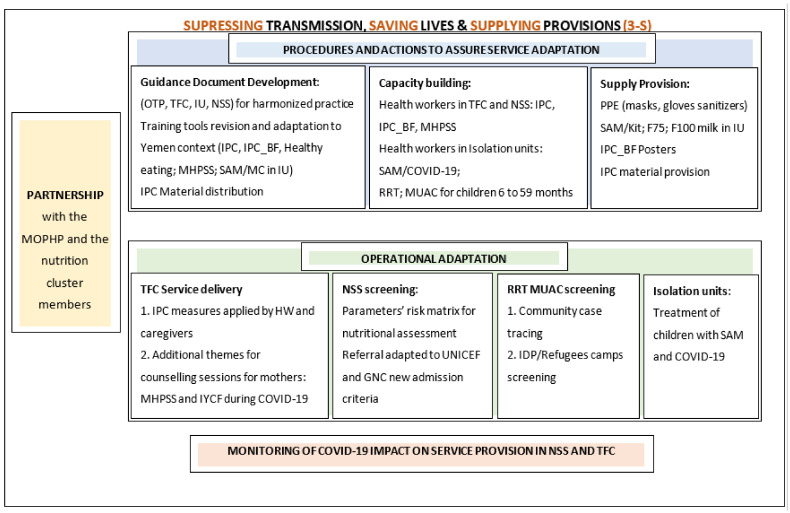
Strategic framework for the adaptation of the nutrition program in Yemen, amidst the COVID-19 pandemic: the 3-S plan. Abbreviations: COVID-19: coronavirus disease 2019; GNC: Global Nutrition Cluster; HW: health workers; IDP: internally displaced person; IPC: infection protection and control; IPC_BF: infection protection and control for breastfeeding; IU: isolation units; IYCF: infant and young child feeding; MC: medical complications; MHPSS: mental health and psychosocial support; MOPHP: Ministry of Public Health and Population; MUAC: mid-upper arm circumference; NSS: Nutrition Surveillance System; OTP: outpatient therapeutic program; PPE: personal protective equipment; RRT: rapid response team; SAM: severe acute malnutrition; TFCs: therapeutic feeding centers; UNICEF: United Nations Children’s Emergency Fund.

Guided by the WHO’s COVID-19 strategic operational planning guidelines to support country preparedness and response [1] where the major pillars of COVID-19 preparedness and response were outlined, a strategic framework was developed for the adaptation of the nutrition program in Yemen. Building on partnership with MOPHP and the nutrition cluster members in Yemen, the plan outlined the priority nutrition steps and actions across the WHO core pillars and was based on a three-pronged strategy: suppressing, saving, and supplying (Three-S). As shown in Figure 1, the programmatic adaptation was structured at various levels, encompassing (1) procedures and actions to assure service adaptation, including the development of guidance documents, capacity building, as well as the supply of provision; (2) operational adaptation which focused on service delivery within TFCs; screening within the NSS; the rapid response team (RRT) for mid-upper arm circumference (MUAC) screening; treatment in isolation units (IU); and (3) the monitoring of COVID-19 impact on service provision in NSS and TFC.

The process of program adaptation was executed through five steps, as summarized in Table 1. As part of the country’s preparedness plan, the adaptation process was initiated in March 2020, before the first COVID-19 case was confirmed in Yemen.

Step 1: In the first step, a situational analysis was conducted between March and April 2020 to identify specific programmatic changes to be temporarily–yet urgently-introduced to ensure the continuity and safety of prevention and treatment services during the pandemic. The situation analysis was undertaken by health system building blocks [26]. The main findings and considerations stemming from this situation analysis are outlined in Table 2.

Step 2: In the second step, and based on findings stemming from the situation analysis, standard operating procedures (SOP), were developed to harmonize practice and assure safe service. SOPs were developed with the aim of ensuring that ongoing nutrition services could follow the necessary infection protection and control (IPC) protocols to reduce the risk of exposure to the virus amongst HWs, as well as service beneficiaries and increase the population trust in the system’s ability to maintain equitable access to essential service delivery throughout this emergency. All SOPs were developed on the main assumption that the COVID-19 National Response Strategy that is currently in place in Yemen, had already established SOPs for screening in healthcare settings (Hospitals and primary healthcare centers (PHC)).

The WHO’s Health Emergency/Nutrition department (WHE/NUT), in partnership with the nutrition cluster and the health cluster, developed SOPs on the safe access to services provided through the NSS, and on inpatient management of severe acute malnutrition with medical complications (in TFC and IU settings) (Table 3). Training tools on IPC, mental health and psychosocial support (MHPSS), and SAM with COVID-19 and healthy eating [27,28,29,30,31,32,33,34,35] were adapted to the Yemeni context. SOPs covered all aspects of service delivery in both outpatient and inpatient settings, including environment, equipment, medical care, food, and interpersonal infection prevention. This includes physical distancing, environmental cleaning, waste management, droplet precaution. Details on the use of appropriate personal protective equipment (PPE) for HWs and in-ward patients were provided, with specific distinctions made for staff working in inpatient wards (stabilization centers), or outpatient screening services.

Step 3: In the third step, a process of consultation was launched to review the SOPs, involving the Health and Nutrition Cluster partners, WHO headquarter (HQ), WHO Office of the Eastern Mediterranean (WHO EMRO), and the Ministry of Health in Aden and Sana’a. The consultations took part essentially in virtual modality amongst the technical partners, and mainly using the Nutrition Cluster and Health Cluster platforms. All SOPs were finalized and submitted to the Ministry of Health (MOH) Aden and Sana’a for review. Consultations with MOH officials took place in person, through several meetings, and comments were duly addressed. The developed SOPs were endorsed by the Directors of Nutrition Departments in Aden and Sana’a MOHs, respectively.

Step 4: In the fourth step, and to operationalize the SOPs in nutrition services, a capacity-building program was designed, developed and rolled out. In collaboration with the nutrition cluster partners and MOPHP, WHE/NUT has developed a comprehensive capacity building program, aimed at enabling HWs to foster their skills in the prevention of infection transmission, procurement of essential supplies, and the revision, innovation, and strengthening of the monitoring strategy. A series of training of trainers (TOT) courses, as well as on the job trainings were conducted on procedures for safe operation of nutrition service during the COVID-19 pandemic, IPC, breastfeeding during COVID-19, healthy eating, as well as mental health and first aid psychosocial support. Skills of medical staff in IU and RRT were also built to assess children for acute malnutrition and treat them in inpatient settings (the latter applies for IU staff). The SOPs developed in Step 3 were integrated into these training programs and were instrumental in guiding the procurement of new and necessary supplies (e.g., PPE, sanitizers, soap, disinfectants, and IPC material). Several training sessions were conducted, including those presented in Table 4. As of December 2020, the total number of training beneficiaries, at different levels, was estimated at 2909, including HWs, nutrition trainers, and RRT team members. Although the outcomes of these trainings in terms of performance assessment on the ground was not performed, the uptick in nutrition screening (as discussed later in Section 5), may serve as an evidence of outcome.

All HWs in the health facility including NSS surveillance, TFC and IU workers have received training on IPC protocols, that include the practice of physical distancing, hand and respiratory hygiene, cleaning and disinfection of equipment, and cleaning of the environment.

Step 5: Programmatic adaptation were incorporated within nutrition services delivery in TFC and IUs and within the NSS. The delivery of nutrition services was integrated within IUs protocols for the appropriate management of U5C children with COVID-19 and severe acute malnutrition, and within TFC to ensure safe continuation of essential nutrition and health services in inpatient wards. Nutrition services, and particularly the assessment of the nutritional status of children aged 6 to 59 months, were also incorporated within community-based surveillance, contact tracing, and RRT protocols, extending this action to internally displaced populations and refugees camps. In addition, and in line with COVID-19 preventive measures, in Yemen, hub focal points (each hub being composed of multiple neighboring governorates) and governmental health offices have been instrumental in disseminating the SOPs on nutritional screening and reporting in nutrition surveillance sites that are established in health facilities (Hospitals and PHCs).

Service adaptation was supported by the WHO through the provision of PPE supplies, including but not limited to masks and gloves and infection prevention supplies, such as hand hygiene sanitizers and disinfectants to ensure full adherence to IPC protocols and prevent or limit infection transmission in health-care settings. The WHO has also equipped IUs with United Nations Children’s Emergency Fund (UNICEF) anthropometric tools, essential medicines, and specialized nutrition products (therapeutic Formula Milk F75, F100 and Ready to Use Therapeutic Food) to treat medical complications associated with severe malnutrition.

## 4. Key Considerations Addressed by the Programmatic Adaptation in Yemen

Nutritional assessment in NSS is normally performed through the measurement of several parameters, including the child’s age, weight, height/length, hemoglobin level, breastfeeding status, oedema, and MUAC measurements. Acknowledging that the assessment of some of these parameters may not be feasible during the COVID-19 pandemic, the SOPs focusing on NSS provided guidance on the identification of the most suitable indicators that may be adopted for nutritional assessment, and how these indicators may be measured during the pandemic. Recognizing that the measurement of nutritional indicators depends on a variety of context-specific factors, different scenarios were put in place. In particular, the possible shortage of IPC material and PPE would necessitate some degree of adaptation. Accordingly, a parameters risk matrix was developed based on two scenarios: (1) Availability of adequate protocols, PPE and IPC material, and disinfectants to sanitize the surfaces of anthropometric tool; and (2) Shortage of adequate protocols, PPE and IPC material, and disinfectants to sanitize the surfaces of anthropometric tools (Table 5).

**Figure 2:**
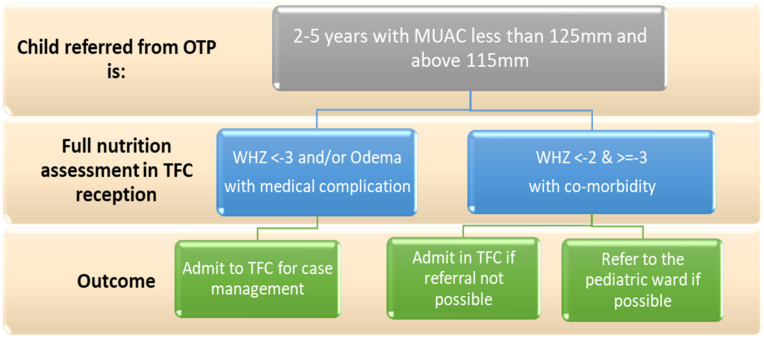
TFC admission criteria for 2–5 years old children with moderate acute malnutrition. Abbreviations: MUAC: mid-upper arm circumference; OTP: outpatient therapeutic program; TFC: therapeutic feeding center; WHZ: weight-for-height Z-score.

For the SOPs on the nutritional care and clinical management of U5C children with SAM in TFC, special considerations were given to children with moderate acute malnutrition (MAM): admission criteria for MAM cases with infection were adapted based on UNICEF global guidance [38]. Accordingly, in hospitals’ emergency rooms (ER) or outpatient departments (OPD) all children 2–5 years referred from the outpatient therapeutic program with MUAC between 115 and 125 mm would be referred to TFC for further investigations [36,39]. In the TFC, the child is assessed for WHZ and oedema, while applying appropriate IPC measures. The child is admitted if WHZ < −3 or has oedema. The child may also be admitted if WHZ is between −2 and −3, with other co-morbidity. If referral was not possible, a child with MAM using MUAC and WHZ, without the presence of oedema, will receive needed medical care but will not receive F75 and F100 (Figure 2).

Children with MAM and COVID-19 will be treated as any other case of suspected COVID-19, and their treatment will take place in the setting of COVID-19 IU. If the child with MAM converts to SAM, then the child will be treated based on the National Protocol for Inpatient Management of SAM with medical complications [38].

IU HWs (medical doctors and nurses) received training on basic principles of inpatient management of SAM with medical complications (SAM/MC), including nutritional assessment, identification of medical complication signs, stabilization, clinical care, and initiation of cautious feeding.

## 5. Monitoring

**Figure 3:**
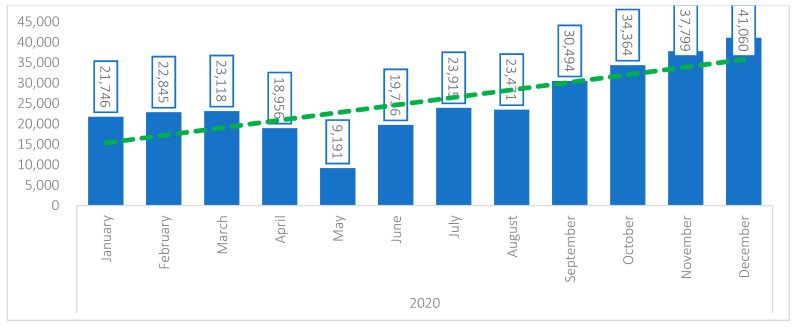
Number of children 0–59 months screened in the 147 NSS operational sites from January to December 2020. Abbreviations: NSS: Nutrition Surveillance System.

The overall strategic adaptation includes a monitoring system, which is regularly revised and adapted to respond to the outbreak and collect, manage, analyze, and report on key indicators to track challenges and progress, plan response, and prioritize actions to improve service delivery [15]. Data up to December 2020 have documented the proportions and trend of indicators tracked through NSS, including underweight (weight for age), acute malnutrition using MUAC, exclusive breastfeeding and oedema [15]. Figure 3 shows the number of U5C children who were screened within the NSS between January and December 2020.

**Figure 4:**
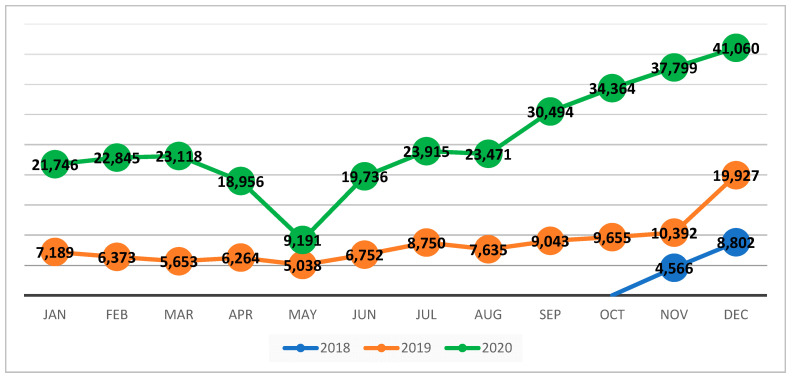
A comparison of the number of children screened within NSS by month in 2018, 2019 and up to December 2020. Abbreviations: NSS: Nutrition Surveillance System.

Figure 4 compared the number of children screened within the NSS by month in 2018, 2019 and 2020, regardless of the total number of NSS, which increased gradually from 42 NSS in 2018 to 147 NSS in 2020. The figure shows that, after a significant dip in the number of children being screened in May 2020 (due to the reduction in all nutrition services utilization during the month of Ramadan, which got further aggravated by the onset of the COVID-19 outbreak in Yemen as of 10 April 2020), the screening went back to previous annual averages, and exceeded those averages in the subsequent months. This improvement can be attributable to the measures put in place (IPC and safe anthropometric assessment) to maintain safe access to surveillance and preserve population trust in the system’s ability to provide safe nutrition services, during the COVID19 pandemic.

**Figure 5:**
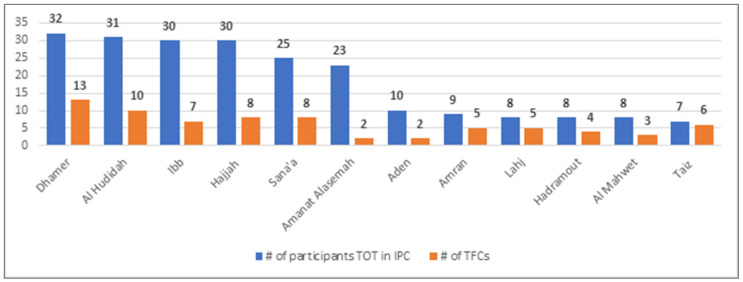
Number of TFCs implementing IPC counseling per governorate and number of participants who participated in training of trainers on IPC. Abbreviations: IPC: infection protection and control; TFCs: therapeutic feeding centers; TOT: training of trainers.

**Figure 6:**
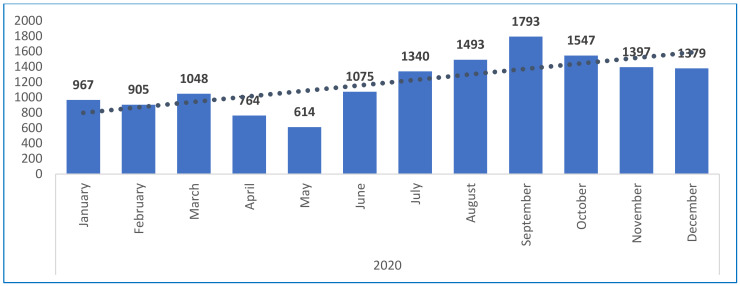
Number of under five children admitted to TFCs in 2020, by month. Abbreviations: TFCs: therapeutic feeding centers.

The number of TFCs in Yemen increased from 90 in May 2020 to 100 in December 2020. As per data available up to December 2020, the number of TFCs implementing IPC counseling was of 73 (Figure 5). Figure 6 shows the number of admitted children in TFCs in 2020, per month, which illustrates an ascending trajectory.

## 6. Discussion

In line with Pillar 9 of the WHO’s COVID-19 Strategic Preparedness and Response Plan [1] and in alignment with the updated WHO guidance on maintaining essential health services during the pandemic [3], this paper reports on the nutrition program adaptations in Yemen to maintain the delivery of essential nutrition services to U5C. To mitigate the impact of COVID-19, protocol adaptations were put in place to ensure safe screening and appropriate nutritional assessment, while protecting children, HWs and caregivers from the risk of cross infections. Guidance documents were developed to harmonize practice in the delivery of nutrition services including NSS, TFC, and IUs. In collaboration with WHO programs involved in COVID-19 response, surveillance, RRT, and infection control pillars, joint programming was used for HWs’ capacity building and service provision. As of September 2020, all service delivery points including nutrition surveillance and TFC have shifted their standard operating procedures into context-specific adaptations that were necessary on the basis of transmission levels, population mobility restrictions, resources, and other local public health measures to respond to and mitigate the effects of the pandemic across the country.

U5C represent a nutritionally vulnerable population group, particularly in humanitarian crises, such as the one currently witnessed by Yemen. Based on the recent food security phase classification (IPC) acute malnutrition report issued in February 2021, approximately 2.3 million U5C in Yemen are projected to suffer from acute malnutrition in 2021 [17]. Malnutrition, in all its forms, exerts significant adverse impact on a child’s physical and cognitive development, and increases the risk of mortality in young children [24]. The WHE/Nutrition program in Yemen has been providing vital nutrition services in the screening, as well as the treatment of malnutrition amongst U5C in Yemen [14,15,16]. However, with the extensive impact of COVID-19 on an already fragile health care system and on people’s behavior in seeking medical care, the delivery of these nutrition services was threatened. In addition, in context of the COVID-19 pandemic, dietary quality and quantity are expected to deteriorate due to the loss of household income and disruptions in food systems (such as the disruption of trade and transport of foods from production to markets), which may further exacerbate malnutrition in young children [3].

In order to ensure safe delivery of essential nutrition services amidst the COVID-19 pandemic in Yemen, a programmatic adaptation of the nutrition program was designed and implemented, as described in this paper. To our knowledge, the documentation of this programmatic adaptation is a novel contribution to the literature. A recent article by Singh et al. (2020) reviewed programmatic adaptations and context specific interventions that are implemented by humanitarian organizations in response to the COVID-19 pandemic [40]. The review categorized these adaptations based on an operational framework that allows locating information by specific humanitarian activity, with one of these activities focusing on nutrition. The review indicated that few countries including Pakistan, South Sudan, Bangladesh have implemented nutrition program adaptations, but without providing any description of these adaptations [40]. A study by Francis and Pegg (2020) [41] in the humanitarian setting of the Niger Delta described an adaptation of the school nutrition program, and its transformation into socially distanced feeding programs, after the forced schools’ closure amidst the pandemic. Additional examples of published programmatic adaptations in the context of COVID-19 have been limited to the adaptation of human immunodeficiency virus (HIV) services in resource-constrained settings during the COVID-19 pandemic [42], adaptations of abdominal transplant programs and patient care practice habits [43], or adaptation in the local farming and animal production sectors to foster food security [44]. The process of adaptation of the nutrition program in Yemen, as described in this paper may, therefore, serve as a prototype for other countries and humanitarian settings to initiate and implement context-specific program adaptations.

The process of adaptation conducted in Yemen has provided frontline HWs with context-specific technical guidance during the challenging COVID-19 pandemic. Supporting HWs with context–tailored technical guidance is crucial, particularly when most available guidance at the global level is primarily normative, and not developed for a humanitarian context or too broad to be adopted in different types of humanitarian settings [40]. Through the documentation of the nutrition programmatic adaptation in Yemen, this paper has revealed the quick reactivity and creativity of frontline humanitarian actors on the ground who are working with vulnerable populations in extremely challenging environments. The amended protocols and the developed guidance documents can serve as a basis for improved nutritional guidance iterations in humanitarian settings amidst the COVID-19 pandemic. The efficiencies and cost savings that may be gained through these adaptations have the potential to preserve investments made in the prevention and control of child malnutrition and to protect beneficiaries and healthcare workers from COVID-19 transmission, while also pushing for innovations that could enhance the effectiveness and efficiency of nutrition programs for years to come.

## 7. Conclusions

In agreement with Pillar 9 of the WHO’s COVID-19 strategic preparedness and response plan [1], this paper reports on the nutrition program adaptations in Yemen to maintain the delivery of essential nutrition services to U5C. To our knowledge, the documentation of programmatic adaptation within the nutrition context is a novel contribution to the literature. The conceptualization, implementation, and monitoring of the service adaptation, as described in this paper may serve as a case study for other countries that intend to undertake similar adaptations in their nutrition program to maintain the delivery of essential nutrition services to vulnerable population groups.

## Figures and Tables

**Table 1:** A five-step process for the introduction of programmatic adaptations.

	Description of Steps
Step 1: Situation analysis	Identification of specific programmatic changes to be introduced to ensure the continuity and safety of prevention and treatment services during the pandemic
Step 2: Development of guidance documents	Development of SOPs and capacity building packages by the WHE/NUT department in partnership with the nutrition cluster and the health cluster
Step 3: Consultation process	Consultation with relevant partners and adoption of the developed SOPs and capacity-building packages
Step 4: Capacity building programs targeting HWs	Design and implementation of a capacity building program targeting HWs in Yemen
Step 5: Incorporation of programmatic adaptation within nutrition services and surveillance	Incorporation of nutrition services (as adapted) within the isolation units, TFC, and RRT for the appropriate management of U5C suffering from SAM and COVID-19, and ensuring safe nutritional assessment in NSS

Abbreviations: COVID-19: coronavirus disease 2019; HWs: health workers; NSS: Nutrition Surveillance System; RRT: rapid response team; SAM: severe acute malnutrition; SOPs: standard operating procedures; TFCs: therapeutic feeding centers; U5C: under-five children; WHE/NUT: WHO’s Health Emergency/Nutrition department.

**Table 2:** Key findings stemming from the situation analysis by health system blocks in Yemen, to be considered in nutrition programmatic adaptation.

Health System Block	Findings Stemming from the Situation Analysis
Technical leadership	- Lack of SOPs, protocols and guidelines for IPC, that are properly adapted to the context of Yemen, the latter being characterized by low resources, fragile health system, ongoing conflict and humanitarian crisis.
Essential supplies	- Lack of appropriate ppe in health facilities for covid-19, including those that serve as stabilization centers and nutrition surveillance sites. - Insufficient access to soap, water, and disinfecting solutions in health facilities to ensure safety and hygiene of equipment.
Health workforce	- Low capacity of HWs to implement IPC measures to contain the spread of the disease. - Repurposing of the health workforce for COVID-19 cases.
Information System	- Gaps in current information system on the monitoring and reporting of services, indicators, and their contributing factors. - Suspension of assessments and surveys which require close physical contact.
Service Delivery	- Reduced access and use of health services due to population movement restrictions. - Deteriorated healthcare-seeking behavior due to fear of transmission. - Weak COVID-19 screening or triage at the health facility entrance. - Suspension of community-level visits and screening by community HWs.

Abbreviations: COVID-19: coronavirus disease 2019; HWs: health workers; IPC: infection protection and control; PPE: personal protective equipment; SOPs: standard operating procedures.

**Table 3:** Description of the developed SOPs within the nutrition program adaptation in Yemen.

Title of the SOP	Description of the SOP
SOP for safe access to services provided through NSS	In addition to the sections on IPC measures and precautions, the NSS SOPs include a section which focuses on the screening process of children 0–59 months for all forms of malnutrition. A risk analysis was conducted to assess the safety of the parameters used to measure child nutritional status (e.g., weight, height, MUAC, Hb levels, oedema). The availability of PPE and IPC material was considered a prerequisite for safely conducting the nutritional assessment of the child.
SOP for nutritional care and clinical management of children aged 0–59 months with SAM in TFCs	The guidance for TFC during COVID-19 is built around the key principle that COVID-19 cases should not be treated in an inpatient ward where non-COVID-19 patients are hospitalized. Therapeutic feeding programs will continue to be provided during COVID-19 with the same technical protocols without any modifications. However, there are some adaptations that are required to ensure safe continuation of the essential health services in inpatient wards according to the stage of care and principally to avoid nosocomial infection (COVID-19 and others), and to reduce the level of anxiety and stigma related to COVID-19. Examples of such modifications include the utilization of the baby friendly space and the IYCF counseling corner in the TFC ward; and the adoption of a modified schedule for the allowed number of children/caregivers per rotation.
SOP for nutritional care and clinical management of children aged 0–59 months, with severe COVID-19 and SAM in IU (COVID Hospitals)	These SOPs detail the work processes that are to be conducted and followed in IU (COVID-19 Hospitals), where children 0–59 months with wasting and medical complications (SAM/MC), who are also COVID-19 positive, are treated as inpatients. Children with wasting and COVID-19 should be treated as inpatient in IUs even if they pass the appetite test. COVID-19 is considered the medical complication.

Abbreviations: COVID-19: coronavirus disease 2019; Hb: hemoglobin; IPC: infection protection and control; IU: isolation units; IYCF: infant and young child feeding; MC: medical complications; MUAC: mid-upper arm circumference; NSS: Nutrition Surveillance System; PPE: personal protective equipment; SAM: severe acute malnutrition; SOP: standard operating procedure; TFC: therapeutic feeding center.

**Table 4:** Capacity building program within the programmatic adaptation in Yemen.

Training on MUAC Assessment	Training on Integrating COVID-19 Measures in Nutrition Programs	Training for Inpatient Management of SAM/MC
To identify acute malnutrition in children aged 6 to 59 months. To capacitate RRT in measuring the mid upper arm circumference in children 6–59 months. To refer the child appropriately.	To capacitate NHW (NSS surveillance and TFC workers) on IPC measures in the context of COVID-19. To enable NHW to assess IPC elements in their facility and make their own IPC plan. To instruct NHW on how to use a facility-based IPC assessment tool. To capacitate NHWs in the correct use of PPE. To enable NHW to detect and refer positive COVID-19 cases and use the referral card. To integrate MHPSS elements in nutrition counseling and Infant feeding guidance in the context of COVID-19.	To capacitate HWs in IUs to treat U5C with confirmed or suspected COVID-19 and SAM. To develop NHW skills in: Assessment of child nutritional status and identification of wasting. Diagnosis and treatment of common medical complications in severely malnourished children and in line with COVID-19 guidance. Feeding the child appropriately during stabilization, transition and rehabilitation phases.

Abbreviations: COVID-19: coronavirus disease 2019; HWs: health workers; IPC: infection protection and control; IUs: isolation units; MC: medical complications; MHPSS: mental health and psychosocial support; MUAC: mid-upper arm circumference; NHW: nutrition health workers; NSS: Nutrition Surveillance System; PPE: personal protective equipment; RRT: rapid response team; SAM: severe acute malnutrition; TFC: therapeutic feeding center; U5C: under-five children.

**Table 5:** Two possible scenarios for nutritional assessment, based on the availability of PPE and IPC material.

Scenario 1: Safe Nutritional Assessment with Availability of PPE and IPC Material	Scenario 2: Safe Nutritional Assessment with Shortage of PPE and IPC Material
This option is implemented if adequate protocols, PPE and IPC material, and disinfectants to sanitize the surfaces of anthropometric tools, including MUAC tapes, are available. The selection of indicators is based on a parameters’ risk analysis. As a result, the following indicators will be assessed during the COVID-19 pandemic: - Underweight: WAZ - Wasting using WHZ and MUAC ^a^ - Nutritionally at-risk using WAZ for infants less than six months ^a^ - Exclusive breastfeeding - Oedema.	This option is implemented if there is a shortage of adequate PPE, IPC material, and disinfectants to sanitize the surfaces of anthropometric tools. MUAC tapes must be disinfected with solution, washed with water and soap, or sanitized after each use. Alternatively, a single-use MUAC tape may be used for each child. The selection of indicators is based on a parameters risk analysis. As a result, the following indicators will be assessed during the COVID-19 pandemic: - Underweight: weight-for-age - Acute malnutrition using MUAC ^b^ - Exclusive breastfeeding - Oedema

^a^: Note about infants 0–6 months. They are considered severely malnourished if they have a weight-for-length Z-score of < −3 Z-score [36], together with breastfeeding or other feeding difficulties. For infants with a length <45 cm, the weight-for-length Z-score has not been determined. The criteria for severe wasting are, therefore, based on weight for-age < −3 Z-score, together with breastfeeding or other feeding difficulties [32]. There are currently no globally established thresholds for MUAC to identify at-risk infants under six 6 months of age. In accordance with existing guidance [37], these infants should be managed in the community if they have no other medical complications and it is assessed that the child can be safely and adequately fed by breastfeeding or another appropriate replacement feed with community support. Avoiding admission for this specific group will reduce their (or their caretaker’s) risk of contracting COVID-19 in a health facility. ^b^: Although height boards are safe to use after sanitization, MUAC may be recommended for children aged 6–59 months in the absence of disinfectant solution. Abbreviations: COVID-19: coronavirus disease 2019; IPC: infection protection and control; MUAC: mid-upper arm circumference; PPE: personal protective equipment; WAZ: weight-for-age Z-score; WHZ: Weight-for-height Z-score.

## Data Availability

Not applicable.

## Abbreviations

COVID-19: coronavirus disease 2019; ER: emergency room; GNC: Global Nutrition Cluster; Hb: hemoglobin; HIV: human immunodeficiency virus; HQ: headquarter; HW: health workers; ICU: intensive care unit; IDP: internally displaced person; IPC: infection protection and control; IPC_BF: infection protection and control for breastfeeding; IU: isolation units; IYCF: infant and young child feeding; MAM: moderate acute malnutrition; MC: medical complications; MHPSS: mental health and psychosocial support; MOH: Ministry of Health; MOPHP: Ministry of Public Health and Population; MUAC: mid-upper arm circumference; NSS: Nutrition Surveillance System; OPD: Outpatient departments; OTP: outpatient therapeutic program; PHC: primary healthcare centers; PPE: personal protective equipment; RRT: rapid response team; SAM: severe acute malnutrition; SOPs: standard operating procedures; TFCs: therapeutic feeding centers; TOTs: Training of trainers; U5C: under-five children; UN: United Nations; UNICEF: United Nations Children’s Emergency Fund; WAZ: weight-for-age Z-score; WHE: WHO’s Health Emergency; WHE/NUT: WHO’s Health Emergency/Nutrition department; WHO: World Health Organization; WHO EMRO: WHO Office of the Eastern Mediterranean; WHZ: Weight-for-height Z-score.
